# Controlled Growth of Sr_*x*_Ba_1−*x*_Nb_2_O_6_ Hopper‐ and Cube‐Shaped Nanostructures by Hydrothermal Synthesis

**DOI:** 10.1002/chem.202000373

**Published:** 2020-04-30

**Authors:** Ola G. Grendal, Inger‐Emma Nylund, Anders B. Blichfeld, Satoshi Tominaka, Koji Ohara, Sverre M. Selbach, Tor Grande, Mari‐Ann Einarsrud

**Affiliations:** ^1^ Department of Materials Science and Engineering NTNU Norwegian University of Science and Technology Sem Sælands vei 12 7491 Trondheim Norway; ^2^ International Center for Materials Nanoarchitectonics (WPI-MANA) National Institute for Materials Science (NIMS) 1-1 Namiki Tsukuba Ibaraki 305-0044 Japan; ^3^ Diffraction and Scattering Division Center for Synchrotron Radiation Research Japan Synchrotron Radiation Research Institute 1-1-1 Kouto Sayo-gun Hyogo 679-5198 Japan

**Keywords:** hopper crystal, hydrothermal synthesis, niobic acid, SBN, Sr_x_Ba_1−*x*_Nb_2_O_6_

## Abstract

Controlling the shape and size of nanostructured materials has been a topic of interest in the field of material science for decades. In this work, the ferroelectric material Sr_*x*_Ba_1−*x*_Nb_2_O_6_ (*x=*0.32–0.82, SBN) was prepared by hydrothermal synthesis, and the morphology is controllably changed from cube‐shaped to hollow‐ended structures based on a fundamental understanding of the precursor chemistry. Synchrotron X‐ray total scattering and PDF analysis was used to reveal the structure of the Nb‐acid precursor, showing Lindqvist‐like motifs. The changing growth mechanism, from layer‐by‐layer growth forming cubes to hopper‐growth giving hollow‐ended structures, is attributed to differences in supersaturation. Transmission electron microscopy revealed an inhomogeneous composition along the length of the hollow‐ended particles, which is explained by preferential formation of the high entropy composition, SBN33, at the initial stages of particle nucleation and growth.

## Graphical abstract

**Take shape**: Controlling the shape and size of nanostructured materials has been a topic of interest in the field of material science for decades. Here Sr_*x*_Ba_1−*x*_Nb_2_O_6_ (*x=*0.32–0.82, SBN) was prepared by hydrothermal synthesis, and the morphology is controllably changed from cube‐shaped to hollow‐ended structures based on a fundamental understanding of the precursor chemistry.

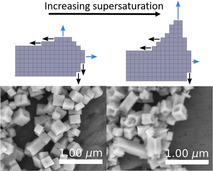

## Introduction

Controlling the size and morphology is an important aspect of material synthesis of functional materials, especially at the micro‐ to nanometer scale, as this is directly linked to material properties.^1^ Thus, understanding and furthermore controlling the underlying mechanisms governing the size and shape of crystalline materials is imperative.^2^ Among a variety of methods for synthesizing crystalline materials, wet chemical synthesis routes, especially hydrothermal, are low‐cost, low‐temperature and scalable methods to prepare functional materials, and have been widely studied to understand crystal growth.^3^ This work focuses on fundamental understanding of crystal growth under hydrothermal conditions.

Intricate structures and morphologies from hydrothermal/solvothermal synthesis have been reported without the use of templates and/or templating agents. This shows the spontaneous formation of complex structures under specific conditions. Some examples are; cubic hopper crystals of Cu_2_O^4^ and PbTe,^5^ hexagonal tubes of NaYbF_4_ 6 and ZnO,^7^ dendrite‐like BaTiO_3_,^8^ tower‐like KNbO_3_,^9^ and morphology‐tuning by doping of α‐Fe_2_O_3_ with various cations.^10^ Almost as many explanations of possible growth and formation schemes exist as there are reported works, even though many of them could possibly be explained by classical growth theory.

From classical growth theory, crystals grow by the addition of monomers (atoms, molecules, or small clusters) from the surrounding solution or vapor phase.^11^ Based on the Berg‐effect,^12^ we know that there is a higher supersaturation at crystal edges and corners, thus the growth is faster at the edges and corners compared to the crystal facets. Still, at low supersaturation, there is an equilibrium between the growth rate of the edges and corners and at the facets, giving crystal shape symmetry reflecting the unit cell of the material with macroscopically flat facets (polyhedral). On the other hand, when the supersaturation is high, this equilibrium can be perturbated, so that the edges and corners are growing at a higher rate than the crystal facets. This is a kinetic effect to reduce the high supersaturation by the formation of a larger surface area and is referred to as an interfacial instability.

The view of crystal growth based on classical growth theory and changes in supersaturation as a function of temperature and pressure can account for the formation of many crystals shapes: the kinetic effect can lead to intricately shaped crystals, sometimes referred to as skeletal crystals, due to an interfacial instability.^13^ Some examples are dendritic crystals observed for ice^14^ and metals,^15^ and hopper crystals observed for some naturally occurring minerals,13a synthetic bismuth,^16^ and NaCl.^17^ Furthermore, for ice crystals, a myriad of naturally occurring morphologies are observed, including hollow structures.^14^ In general, with increasing interfacial instability, the degree of complexity of the final morphology increases (polyhedral→hoppers→dendrites). However, classical growth theory is derived for one‐component or simple binary systems, and the complexity must necessarily increase for wet chemical synthesis with the addition of solvent‐crystal interactions, surfactants and mineralizers. This additional complexity gives an extra dimension for crystal engineering.^18^ As an example for such growth of shaped crystals under complicated hydrothermal conditions, we selected a Sr_*x*_Ba_1−*x*_Nb_2_O_6_ (SBN100*x*) system, a ferroelectric tungsten bronze (TTB) with a second‐order Jahn–Teller polarization mechanism.^19^ This system has received considerable attention due to electro‐optical properties^20^ and cation disorder.^21^ We have previously reported a template‐free hydrothermal synthesis of SBN (*x=*0.2–0.6).^22^ With a precursor slurry corresponding to SBN40 we observed formation of cube‐shaped particles (ca. 500×500 nm) for reactions at 300 °C, whereas for SBN20 we observed the formation of elongated hollow particles (apparently hollow by scanning electron microscopy, SEM) for the same reaction conditions. Similar hollow structures were also observed for higher Sr fractions when the reaction temperature was decreased. A higher specific surface area (m^2^ g^−1^) is expected for the hollow particles compared to the cubes, which is interesting with respect to the photocatalytic activity of SBN,^23^ meriting further investigation into the formation and growth of these structures.

In this work we complement our previous in situ study^22^ with new experiments and characterization, in addition to discussing some of the previous findings in a new setting (explaining the formation of hopper‐crystals). The hollow structures were characterized by transmission electron microscopy (TEM) with a combination of high‐angle annular dark‐field scanning TEM (HAADF‐STEM) and energy‐dispersive X‐ray spectroscopy (EDS). The amorphous structure of the Nb‐acid used as precursor was investigated by synchrotron X‐ray total scattering and pair distribution function (PDF) analysis, which gives valuable insight into the early formation stages of SBN. We show, that based on a fundamental understanding of the precursor chemistry, we can control the growth mechanism and thus the particle morphology of SBN, by tuning the Nb‐supersaturation of the system, both with chemical (Sr:Ba ratio) and kinetical means (reaction temperature).

## Experimental Section

**Synthesis**: Nominal composition Sr_*x*_Ba_1−*x*_Nb_2_O_6_ (*x=*0.2 and 0.4) was prepared following a previously described route.^22^ Strontium nitrate (Sigma–Aldrich, Oslo, Norway, 99.995 %) and barium nitrate (Sigma–Aldrich, Oslo, Norway, 99.999 %) were mixed with a niobic acid (Nb‐acid) aqueous dispersion and pH was adjusted to 12.4 with aqueous ammonia solution (Sigma–Aldrich, Oslo, Norway, 25 wt % solution). The Nb‐acid was prepared by precipitation from an ammonium niobate (V) oxalate hydrate (Sigma–Aldrich, Oslo, Norway, 99.99 %) aqueous solution with addition of ammonia solution to pH ca. 11. The precursor slurries were prepared for each experiment by first weighing out Nb‐acid, then adjusting the pH to 12.4 by adding ammonia solution, before adding the stoichiometric amounts of nitrates and diluting with water to a total volume of 5 mL. This gave a final Nb‐concentration of about 0.25m.

The reactions in this work were performed in a tube coil synthesis setup, as described by Skjærvø et al.^24^ The setup consists of a 316 L steel tube coil that is filled with the precursor slurry and connected to a high‐pressure liquid chromatography (HPLC) pump (Shimadzu LC‐10ADVP, Shimadzu Corporation, Kyoto, Japan) with Swagelok tubes and fittings, using distilled water as pressure media. The coil was heated in a fluidized sand bath (Omega FSB‐3, Omega Engineering, Norwalk, USA) and the temperature was controlled using a PID controller. An overview of the synthesis parameters for the experiments conducted are presented in Table 1. The nomenclature is SBNXX TYYY Z, where XX refers to the Sr mole fraction in the precursor slurry times 100, YYY the reaction temperature and Z the reaction time for the coil experiments and “in” for the in situ experiments (described in the next paragraph). After the reaction, the samples were collected, washed with distilled water by centrifugation and decanting, before drying at 105 °C for about 12 h.

**Table 1:** Overview of experiment names, temperature and reaction time for the coil and in situ synthesis experiments. Also included is the final product and morphology. The pressure for all experiments was 200 bar.

Name^[a]^	Temperature [°C]	Reaction time [h]	Final product	Setup used	Final morphology
SBN40 T300 1 h	300	1	SBN	Coil	Cubes
SBN40 T300 6 h	300	6	SBN	Coil	Cubes
SBN40 T200 1 h	200	1	Amorphous, SBN	Coil	N/A
SBN40 T200 6 h	200	6	SBN	Coil	Rods
SBN20 T300 1 h	300	1	SBN	Coil	Hollow‐ended/Hoppers
SBN20 T300 6 h	300	6	SBN	Coil	Hollow‐ended/Hoppers
SBN40 T300 in	300	0.6	SBN	In situ	Cubes
SBN40 T225 in	225	1.3	SBN	In situ	Cubes
SBN40 T200 in	200	2.4	SBN	In situ	Hollow‐ended/Hoppers
SBN30 T300 in	300	0.6	SBN	In situ	Cubes
SBN30 T225 in	225	1.4	SBN	In situ	Hollow‐ended/Hoppers
SBN30 T200 in	200	2.6	SBN	In situ	Hollow‐ended/Hoppers
SBN20 T300 in	300	0.5	SBN	In situ	Hollow‐ended/Hoppers
SBN20 T225 in	225	2.2	SBN	In situ	Hollow‐ended/Hoppers
SBN20 T200 in	200	4.1	SBN	In situ	Hollow‐ended/Hoppers

[a] The nomenclature is SBNXX TYYY Z, where XX refers to the Sr mole fraction in the precursor slurry times 100, YYY the reaction temperature and Z the reaction time for the coil experiments and “in” for the in situ experiments.

The samples referred to as in situ experiments in this work were performed in our previous work,^22^ and are included here for completeness (samples in Table 1 ending with “ in”). In short, the reaction vessel for these experiments was a sapphire capillary heated with a hot air stream instead of the steel coil and fluidized sand bath used for the ex situ experiments. The same HPLC pump as described above was used for pressurization. The experiments were conducted at the Swiss‐Norwegian Beamlines (BM01), European Synchrotron and Radiation Facility (ESRF), Grenoble, France, and reaction time was optimized for each experiment for best utilization of the allocated beam time. The key differences between the coil setup used in this work and the in situ setup used previously is the reaction vessel (steel tube coil vs. single‐crystal sapphire capillary) and heating rate (ca. 20 s vs. ca. 1 min to reach set point temperature). Further experimental details for the in situ experiments and the in situ setup are reported in previous papers.^22^, ^25^ The coil, and the in situ setups show comparable results with similar reaction conditions as presented in Figures S1 and S2 in Supporting information.

**Characterization**: Phase purity was investigated by X‐ray powder diffraction (XRD) using a Bruker D8 Advance Da‐Vinci equipped with a LynxEye detector working in Bragg–Brentano geometry. Diffraction patterns were recorded with Cu_Kα_ radiation (*λ*=1.5406 Å), a step size of 0.013° and an integration time of 0.75 s using a variable divergent slit.

Scanning electron microscopy (SEM) was performed on a field emission FEI APREO SEM. An in lens secondary electron detector with an acceleration voltage of 5 keV and a current of 25 pA was used. TEM was performed on a double‐corrected JEOL JEM ARM200F with a cold field emission gun. The acceleration voltage was set to 200 kV. The beam convergence angle was 27 mrad and the HAADF‐STEM images were acquired using a collection angle of 51–203 mrad. EDX spectra were acquired using a JEOL Centurio SDD detector (solid angle 0.98 sr), using an energy‐dispersion of 10 eV per channel. The EDS data were analyzed using *HyperSpy* (version 1.4.1).^26^ Samples for both SEM and TEM were prepared by making a dispersion of the dried particles in distilled water using ultrasound bath. The dispersions were then dropped onto the sample holder and let dry for 12–24 h. The sample holders were a FIB stub and a holey carbon copper grid for SEM and TEM, respectively.

Specific surface area (m^2^ g^−1^), was measured by nitrogen adsorption and calculated using the BET method for SBN20 T300 1 h and SBN40 T300 1 h using a Tristar 3000 (Micrometrics Instrument Corporation, Norcross, USA). The samples were degassed at 200 °C for 17 h to remove adsorbed water prior to measurements.

X‐ray total scattering data for pair distribution function (PDF) analysis was collected on BL08W at SPring‐8, Japan using a 16 inch PerkinElmer XRD 1621 CN3 ES series flat panel detector and *λ*=0.10765 Å (115 keV) with a sample to detector distance of ≈53 cm.^27^ Sample to detector distance, and geometry of the setup were calibrated using a NIST CeO_2_ standard. X‐ray total scattering was collected for the as‐prepared Nb‐acid dispersion, the same Nb‐acid dispersion vacuum‐dried at room temperature (this was done for improving the scattering signal) and the fully crystalline samples from the coil experiments (see Table 2). The vacuum dried powder sample and SBN samples were packed in Kapton tubes (OD 1.05 mm, ID 1.00 mm, Goodfellow, England) for the X‐ray measurements. A measurement was done with an empty Kapton tube with same dimensions for background subtraction. Wider tubes (OD 1.90 mm, ID 1.80 mm) were used for the Nb‐acid dispersion experiments, and a Kapton tube with the same dimensions filled with a 1 % aqueous ammonia solution (the dispersions consisted of Nb‐acid in a 1 % aqueous ammonia solution having a Nb‐concentration of ca. 0.9 m) was used for background subtraction. Data treatment (masking parasitic regions like beam stopper, and integration from 2D‐ to 1D‐data) was done using *pyFAI* (version 0.17.0)^28^ and *Jupyter Notebook* (version 5.7.8).^29^ *xPDFsuite* 30 was used for background subtractions and corrections, before Fourier transformation into PDF using a Q range up to 15 and 29.5 Å^−1^ for the Nb‐acid samples and SBN, respectively (note that Q is the moment transfer of scattering particle). *TOPAS* (Bruker AXS version 6) in launch mode was used to fit models to the resulting PDFs, using *jEdit* (version 4.3.1) as the text editor for writing macros for *TOPAS*.^31^ The structural model used for SBN, is described in Supporting Information.

**Table 2:** Average Sr‐fractions obtained from PDF analysis of the X‐ray total scattering data of the fully crystalline materials from the coil synthesis.

Name	Sr‐fraction
SBN40 T300 1 h	0.35
SBN40 T300 6 h	0.35
SBN40 T200 6 h	0.33
SBN20 T300 1 h	0.25
SBN20 T300 6 h	0.24

## Results

**Figure 1:**
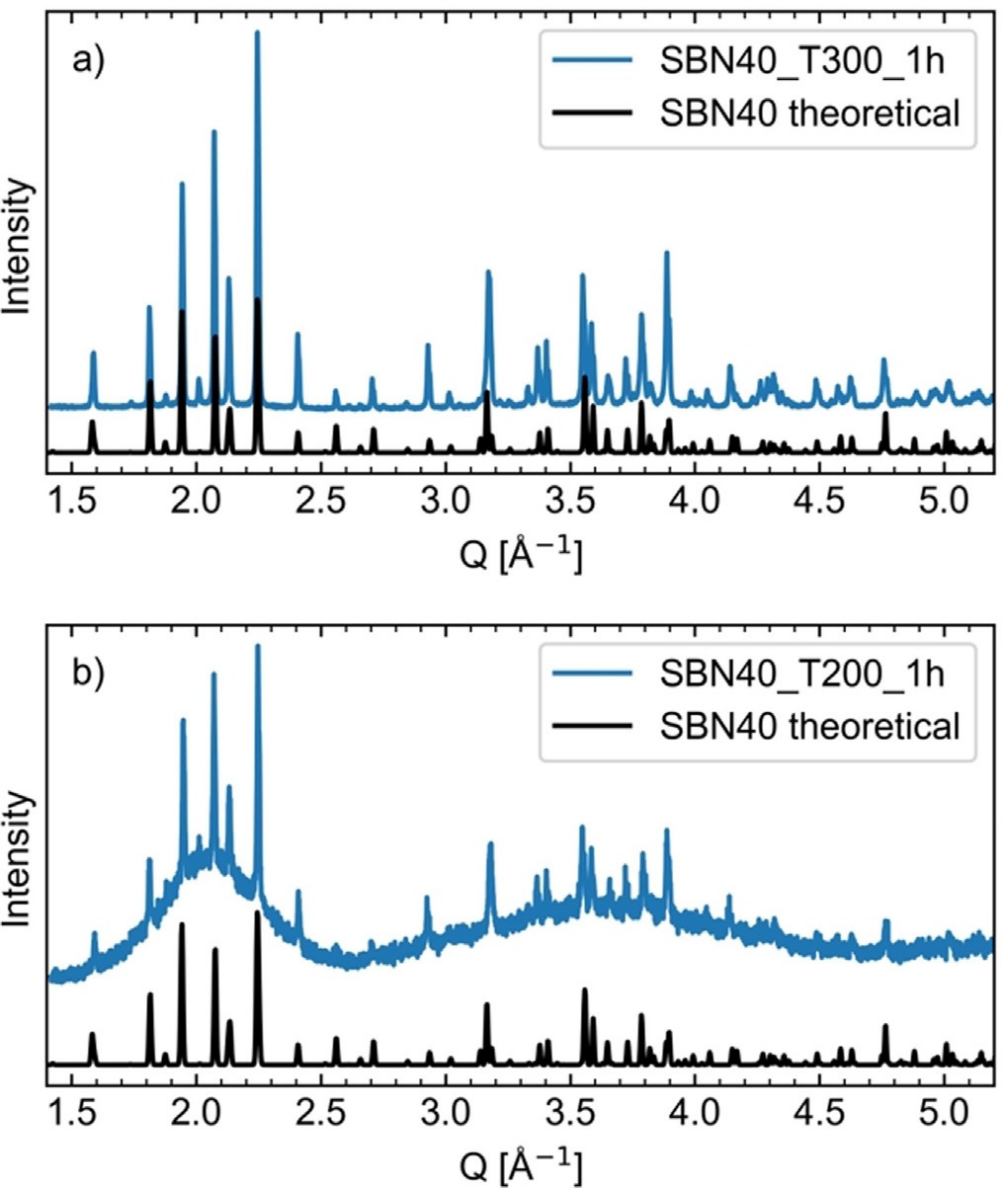
Normalized XRD patterns of a) SBN40 T300 1 h and b) SBN40 T200 1 h. In black is a theoretical diffraction pattern for SBN40 based on Carrio et al.^32^

All the experiments yielded phase pure SBN, as represented by the X‐ray diffractogram of SBN40 T300 1 h shown in Figure 1 a). All materials show high crystallinity, except SBN40 T200 1 h where two broad features showing the presence of amorphous phase as seen in Figure 1 b). This is in good agreement with our in situ findings, where this reaction took over 2 h to complete, whereas for the other experiments conducted in this work the reactions was completed well within 1 h.^22^ The X‐ray diffraction patterns for the coil synthesis experiments and the PDFs are presented in Figure S3 and Figure S4, respectively, in the Supporting Information. The Sr‐fractions (referring to *x* in the chemical formula Sr_*x*_Ba_1−*x*_Nb_2_O_6_) obtained from the PDF analysis are presented in Table 2, while the remaining results are presented in Table S1.

### Structure of the Nb‐acid precursor

**Figure 2:**
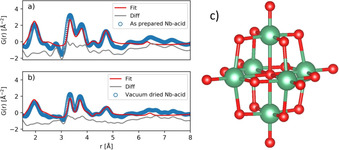
PDF data fitted with a cluster model of the Lindqvist‐ion for the as‐prepared and the vacuum dried Nb‐acid in panel a) and b), respectively. c) Model of the Lindqvist‐ion ([Nb_6_O_19_]^8−^), made using VESTA^35^ based on Ref. 33b. Nb‐ and O‐atoms are green and red, respectively.

PDFs of both the as‐prepared Nb‐acid used as precursor and the vacuum‐dried Nb‐acid are presented in Figure 2 a and b. Except for some intensity differences, and a small shift of the 3.7 Å peak (towards higher *r*‐values for the as‐prepared Nb‐acid), the two PDFs look similar. That is, the vacuum drying did not change the amorphous structure of the Nb‐acid. Four main features can be observed in both PDFs, a broad peak at 1.9 Å, two close peaks at 3.3 and 3.7 Å, and a peak at 4.7 Å. The 1.9 Å peak is assigned to Nb−O atomic pairs in an octahedral configuration, the 3.3 and 3.7 Å peaks are assigned to Nb−Nb pairs of edge‐sharing octahedra, and the 4.7 Å is assigned to the second coordination sphere of Nb−Nb pairs of edge‐sharing octahedra. In addition to these apparent peaks, some weaker correlations are observed between 5 and 8 Å. No correlations are observed beyond 8 Å, indicating only short‐range order.

The Lindqvist‐ion, also known as hexaniobate ([Nb_6_O_19_]^8−^), is a well‐known polyoxometalate appearing in aqueous Nb‐chemistry under alkaline conditions.^33^ The Lindqvist‐ion can be described as a superoctahedron, consisting of 6 edge‐sharing NbO_6_ octahedra, see Figure 2 c, with reported characteristic features at about 2.0 Å (Nb−O distance in octahedra), 3.4 Å (Nb−Nb distance between edge‐sharing octahedra) and 4.7 Å (Nb−Nb distance diagonal),^34^ and is thus a candidate for the structure. A fit of a cluster model of the Lindqvist‐ion to our PDF data is presented in Figure 2 a and b. To fit the peaks at 3.3 and 3.7 Å, the Lindqvist‐ion has been stretched slightly along one of its three equivalent axes in our model. It is known that alkali metal‐ions coordinate around the Lindqvist‐ion,33a and it is not unlikely that the NH_4_^+^‐ion can do the same. If the NH_4_^+^‐ion only coordinates on some of the Lindqvist‐ion faces (as is the case for Li/Na/K), this could explain the perturbation, or stretching of the Lindqvist‐ion. It can be seen that the four main features (1.9, 3.3, 3.7, and 4.7 Å) are present, and that the intensities are fitted fairly well with the Lindqvist‐ion model. It therefore seems clear that a Lindqvist‐like ion is the main motif or building block in the Nb‐acid used. The weaker correlations between 5 and 8 Å could be a signature of coordinated NH_4_^+^‐ions (which are not included in our model). Another explanation could be a partly condensation of Lindqvist‐ions, which could then also contribute the peak at 3.7 Å, if the Lindqvist‐ions were linked through corner‐sharing NbO_6_ octahedra with an Nb‐O‐Nb angle of 154° (calculated from the observed distances). For example, the decaniobate is a well‐known (two edge‐sharing Lindqvist‐ions, [Nb_10_O_28_]^6−^) polyoxometalate, although normally stable only at lower pH values than used in this work.33c

### Characterization of size and morphology

**Figure 3:**
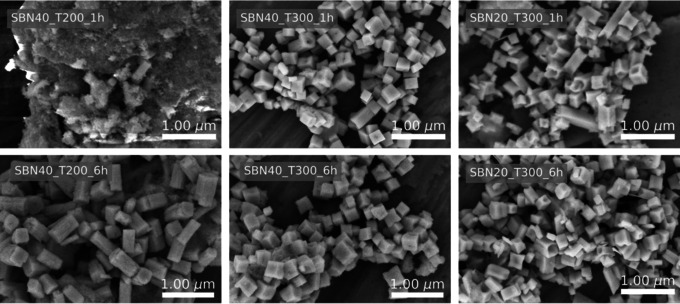
SEM images of SBN particles synthesized with the coil setup with varying Sr fraction in the precursor slurry, reaction temperature and reaction time.

SEM images of the materials prepared by the coil synthesis are presented in Figure 3. For SBN40 with a reaction temperature of 300 °C cube‐shaped particles with a size of about 500 nm are observed for both reaction times. By decreasing the reaction temperature from 300 to 200 °C with a reaction time of 6 h the particles become more elongated, with a cross section with similar dimensions as the cubes formed at 300 °C. In the case of SBN40 T200 1 h, large amorphous agglomerates are observed, which is in good agreement with the XRD pattern. In addition, elongated, apparent non‐amorphous features like rod‐shaped SBN could be observed in the amorphous agglomerates. Decreasing the Sr fraction in the precursor slurry yields elongated particles with a hollow or hollow‐ended structure. Even though some small dimples can be observed on the facets of the SBN40 materials, it is clear that the hollow structures appear upon lowering the Sr content in the precursor slurry. The hollow features for SBN20 at 300 °C become less pronounced with increasing reaction time, making the particles more cube‐like. The surface areas measured for SBN20 T300 1 h and SBN40 T300 1 h were 14.5±0.1 and 15.4±0.1 m^2^ g^−1^, respectively.

**Figure 4:**
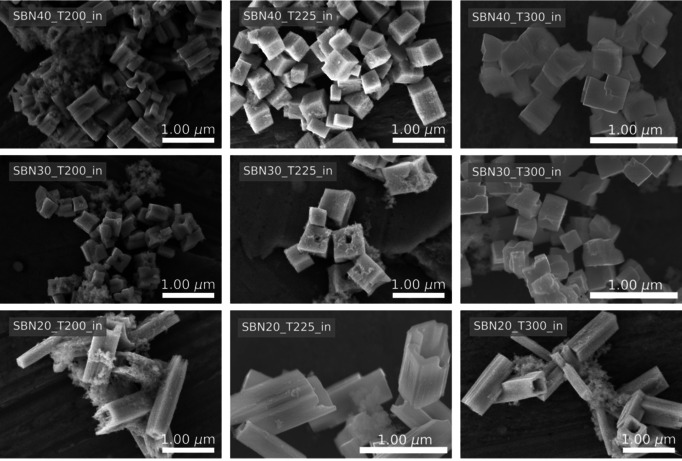
SEM images of SBN particles synthesized with the in situ setup with varying Sr fraction in precursor slurry, reaction temperature and reaction time.

**Figure 5:**
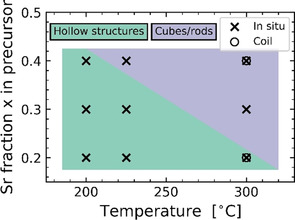
Phase diagram for the formation of the hollow structures as a function of reaction temperature and Sr fraction in the precursor slurry. Circles and crosses indicate experiments with the coil and in situ setup, respectively.

SEM images of the materials prepared by the in situ setup are presented in Figure 4. Cube‐shaped particles with a size of about 500 nm are observed for SBN40 at 300 °C, and with decreasing temperature to 225 °C we observe a slight elongation of the particles. At 200 °C, a morphology similar to the hollow structure of SBN20 is observed. For SBN30 a similar trend is observed as for SBN40, with cube‐shaped particles at high temperature, and more hollow‐like structures with decreasing temperature. In case of SBN30, the transition from the cube‐shaped particles to the hollow structures occurs at a higher temperature than for SBN40, with the formation of hollow structures even at 225 °C. Hollow structures are clearly present at all temperatures for SBN20 from the in situ setup. In addition to the pronounced effect of composition, the in situ experiments also demonstrate a clear temperature effect, where a lower reaction temperature promotes the formation of the hollow structures. These findings are summarized in the phase diagram in Figure 5, illustrating the formation of hollow structures as a function of Sr‐fraction in the precursor slurry and the reaction temperature.

### Characterization of hollow structures with TEM

**Figure 6:**
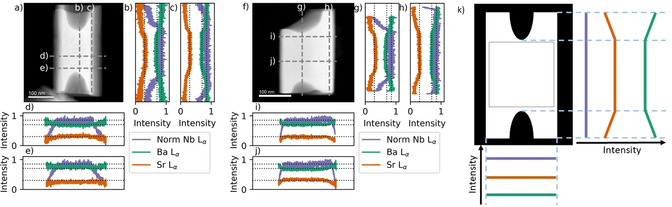
High‐angle annular dark field (HAADF‐STEM) images of two hollow ended rods from SBN20 T300 1 h in a) and f). Grey dashed lines in a and f shows where the EDS line scans were performed, with the corresponding EDS data in panels b), c), d), e), g), h) i) and j). Black dotted lines in panels b), c), d), e), g), h) i) and j) are guides for the eyes, plotted at 0.3, 0.7, and 0.85, respectively. For Nb L_α_ the data have been normalized to the maximum value, while Ba L_α_ and Sr L_α_ have been normalized to the sum of Ba and Sr counts with the assumption that Nb L_α_ is constant where there are no thickness gradients. In k) a schematic representation of the intensity distributions, Nb L_α_, Sr L_α_ and Ba L_α_ are shown. Indicated is the area in the center of the particles with a homogenous composition.

High‐angle annular dark‐field (HAADF) scanning TEM of two representative particles from SBN20 T300 1 h are presented in Figure 6 (the corresponding bright field images are presented in Figure S5 in Supporting Information). Contrast in HAADF‐STEM is directly linked with the thickness of the particles assuming constant average Z number. From these images it is clear that the particles are not hollow all the way through, but consists of a solid center with hollow ends (intensity profiles across the images are presented in Figure S5).

EDS scans for Ba L_α_, Nb L_α_ and Sr L_α_ along four different lines on the particles, with a schematically representation of the compositional variation, are presented in Figure 6 k. Along the horizontal direction no compositional differences are observed, neither when scanning across the middle of the particle, (Figure 6 d and j) nor towards the ends with the hollow structure (Figure 6 e and i). In the vertical direction, little to no compositional difference is observed in the center of the particles (Figure 6 b, c, g and h), but a significant decrease in Sr content is observed towards the hollow ends, while the Nb content stays constant throughout. A minor increase in the Ba content is evident with the decrease in the Sr content. In the regions where the Sr and Ba counts are close to constant (center of particles), the Sr:Ba ratio is approximately 0.3:0.7.

From electron diffraction (Figure S6 in Supporting Information) it is shown that the length of the hollow structures is along the crystallographic [001]‐direction, and that the [1 0 0]‐ and [0 1 0]‐directions (these are equivalent in the tetragonal unit cell) are pointing towards the edges. Hence the facets are normal to the [1 1 0]‐direction (full description in Figure S6).

## Discussion

Single phase SBN, possessing a hollow microstructure was successfully synthesized using a hydrothermal route. It was shown that the structures were not hollow throughout, but consisted of a solid core with hollow ends. Furthermore, the morphology is highly tunable with the synthesis parameters (reaction temperature and Sr fraction in the precursor slurry) from the hollow‐ended structures to cubes and rod‐like structures. The hollow‐ended structures are observed only for low Sr‐fractions (SBN20) at 300 °C, but for a wider range of Sr‐fractions (SBN20 to SBN40) at 200 °C. It is clear that both a low Sr fraction in the precursor slurry and a low reaction temperature promotes the formation of the hollow‐ended structures. The hollow‐ended structures possess a high specific surface area (SBN20 T300 1 h), but not higher than cubes (SBN40 T300 1 h). This is in good agreement with the macroporous nature of the hollow‐ended structures, and the limitations of nitrogen adsorption and the BET method to measure the effect of macropores.^36^

### Growth mechanism of hollow‐ended SBN

The hollow‐ended structures presented here are similar to snow crystals14a and olivine in the SiO_2_‐Al_2_O_3_‐CaO‐MgO system from glass cooling experiments,^37^ and can be referred to as a form of hopper crystal. This crystal shape indicates formation under high supersaturation and rapid growth relative to the conditions giving cube‐ or rod‐shaped crystals. As Nb‐species have the lowest solubility of the three cations (Nb^5+^, Sr^2+^ and Ba^2+^), we propose that the concentration of Nb will be the determining factor for the supersaturation in our system, and the discussion will be focused around this.

If we first consider the reactions at 300 °C with varying Sr‐fraction in the precursor slurry, only SBN20 is forming hollow‐ended structures. Hence the supersaturation increases with decreasing Sr‐fraction, since the hopper‐growth mechanism is promoted only at higher supersaturations. Knowing that our Nb‐acid precursor consists of motifs of the Lindqvist‐ion,33a, 33d we infer from the observations that the solubility of this ion increases with decreasing Sr fraction, giving the higher supersaturation. Normally, the solubility of salts increases with increasing size difference between the ions (Lindqvist‐ion is large, and therefore closer in size to Ba^2+^ than Sr^2+^), so one would expect the Lindqvist‐ion to be more soluble with higher Sr fractions from this simple consideration. However, for the Lindqvist‐ion the opposite trend is observed for salt‐complexes formed with alkali metal ions. The solubility of these salt‐complexes increases going down in the periodic table, for example, solubility increases with decreasing size difference (solubility increases Li<Na<K<Rb<Cs),33a, 38 just as indicated for the alkaline earth cations in this work. The Sr:Ba fraction is thus observed to give chemical control over the growth mechanism of SBN, where the system can be pushed into the hopper‐growth regime with decreasing Sr‐fraction. Similar effects have been observed for NaCl crystals growing from an aqueous solution, where an abrupt change from layer‐by‐layer growth forming cubes to a hopper‐growth mechanism was observed as a function of increasing supersaturation estimated at the time of nucleation,^17^ and crystallization of struvite (NH_4_MgPO_4_ **⋅**6 H_2_O) where morphology control was obtained by controlling the supersaturation of the system.^39^ If we now look at the reactions at 200 °C, the hollow‐ended structures were observed also for SBN40 and SBN30 in addition to SBN20. Using the same argument as above, SBN20 has a relatively higher supersaturation than SBN30 and SBN40 also at 200 °C because of the lower Sr‐fraction. We propose that with the lower reaction temperature and thus slower kinetics, a larger supersaturation is needed to overcome the nucleation barrier. This shifts the nucleation to occur at a relatively higher supersaturation for all compositions, making SBN20, SBN30 and SBN40 all nucleate in the hopper‐growth region. In addition to the chemical control, the growth mechanism can therefore be controlled also by the kinetics via the reaction temperature.

**Figure 7:**
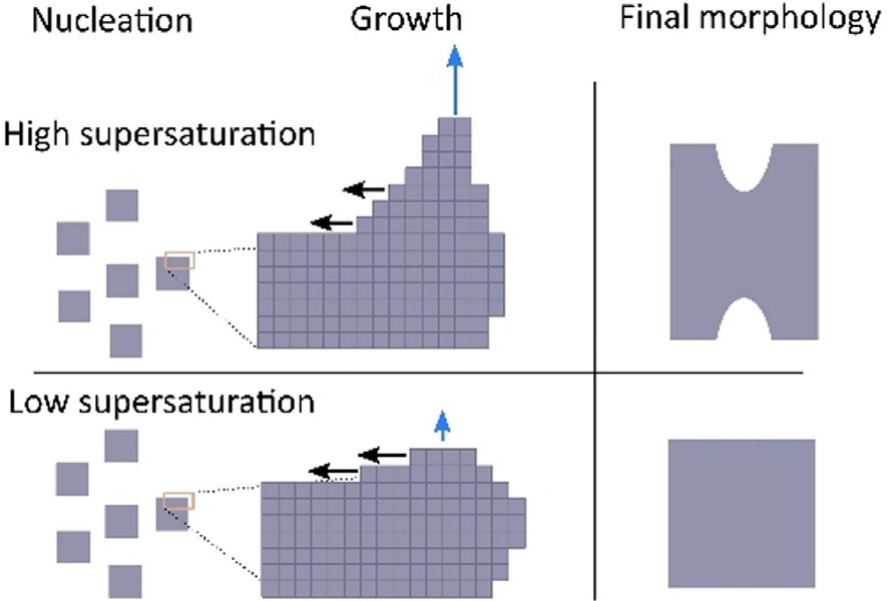
Schematic illustration of the effect of supersaturation on the growth mechanism of SBN. Blue arrows indicate the nucleation rate of new layers, and black arrows the completion of these layers. At high supersaturation the nucleation rate is much higher than the rate of completion, leading to higher growth rates at the edges and corners.

Some additional general comments to the mechanism of hopper growth are worth including. Hopper growth occurs under high supersaturation, and the initial rapid growth is limited by the surface area of the growing particles. The hopper growth mechanism is a way for the system to quickly increase the surface area of the growing particles, making the growth diffusion‐limited.^17^ A rapid initial growth of SBN was observed for the in situ experiments,^22^ in good agreement with the proposed hopper growth mechanism. A growth rate order of 3 is reported for the hopper growth of the NaCl crystals which is comparable to the values around 2.5 that we reported for SBN,^22^ further supporting the proposed hopper‐growth mechanism in this work. A general scheme for the growth mechanism is presented in Figure 7.

For the growth of the cube‐ and rod‐shaped particles observed at high temperatures and/or high Sr‐fractions in the precursor slurry we propose a classical layer‐by‐layer growth mechanism. The change from cube‐ to more rod‐shaped particles observed for SBN40 with decreasing reaction temperature is rationalized with slower kinetics at lower temperatures, promoting the formation of the most stable shape (Wulff construction).^22^

### Composition of hollow‐ended rod‐shaped SBN

It is clear from the EDS line scans that there is a change in composition (Sr:Ba ratio) along the vertical direction of the rods, where the Sr‐fraction is decreasing towards the edges. It is interesting to notice that this is not a gradual decrease from the middle of the rods, but it has a sharp onset in the region close to where the hollow structures start. In the horizontal direction, little to no change in composition is observed. The non‐hollow center of the particles has a close to homogenous composition, with a Sr fraction of ≈0.3 (based on the relative intensities of Sr and Ba). In our previous work, the average composition of the SBN formed had Sr‐fractions of 0.35±0.1 for precursor slurry compositions ranging from 0.2 to 0.5.^22^ In this work we report average Sr‐fractions of ≈0.25 and ≈0.34 for precursor solution of 0.2 and 0.4, respectively. SBN33 is the composition with potentially the highest configurational entropy,^21^, ^40^ making it the energetically favored composition from an entropy point of view. Nonetheless, from our data (EDS and PDF analysis in this work and Rietveld refinement in our previous work^22^), it seems clear that SBN33 is nucleating and growing at the initial stages of the reaction, irrespective of the composition in the precursor slurry, for the syntheses described here. In case of SBN20, there is a limit on how much SBN33 can form, before Sr becomes significantly depleted, and SBN with a higher Ba fraction is formed, explaining the decrease in Sr‐fraction towards the edges of the hollow‐ended structures.

## Conclusions

We have investigated the growth mechanisms for cube‐ and hollow‐ended shaped particles of SBN made by hydrothermal synthesis. We show that the morphology can be tuned with the Sr:Ba ratio in the precursor slurry and the reaction temperature. A structure strongly related to the Lindqvist‐ion for the Nb‐acid precursor was demonstrated by X‐ray total scattering. Based on this knowledge, we could rationalize both the composition and temperature dependence on the formation of the hollow‐ended structures based on relative changes in supersaturation, and a change from layer‐by‐layer to a hopper growth mechanism at high supersaturations. The hollow‐ended SBN structures are more Sr deficient towards the edges in the long direction, explained by a preferential formation of SBN33 at the initial stages of the reaction.

## Conflict of interest

The authors declare no conflict of interest.

## Supporting information

As a service to our authors and readers, this journal provides supporting information supplied by the authors. Such materials are peer reviewed and may be re‐organized for online delivery, but are not copy‐edited or typeset. Technical support issues arising from supporting information (other than missing files) should be addressed to the authors.

Supplementary
