# Supplementary material for: Controlled Growth of SrxBa1−xNb2O6 Hopper‐ and Cube‐Shaped Nanostructures by Hydrothermal Synthesis
Source: Chemistry. 2020 Apr 30;26(42):9348–55. doi: 10.1002/chem.202000373 (PMC7496942; doi:10.1002/chem.202000373)
Supplement: Supplementary file 1 — Supplementary [file CHEM-26-9348-s001.pdf]

Chemistry–A European Journal

Supporting Information

Controlled Growth of $Sr_xBa_{1-x}Nb_2O_6$ Hopper- and Cube-Shaped Nanostructures by Hydrothermal Synthesis

Ola G. Grendal,[a] Inger-Emma Nylund,[a] Anders B. Blichfeld,[a] Satoshi Tominaka,[b] Koji Ohara,[c] Sverre M. Selbach,[a] Tor Grande,[a] and Mari-Ann Einarsrud*[a]

Comparison between SBN40_T300_1h (coil) and SBN40_T300_in (*in situ*)

The main differences between the coil and *in situ* setup is the heating rate (higher for the *in situ* setup), reaction vessel and the reaction time, which is optimized for each *in situ* experiment to maximize the allocated beam time. Comparing the XRD patterns and SEM images for SBN40_T300 from both the coil (SBN40_T300_1h) and *in situ* (SBN40_T300_in) setups in Figure S1 and S2 respectively, similar results are obtained, at least for similar reaction times, despite the difference in heating rate. From XRD, phase pure SBN is observed for both setups, and qualitatively the diffraction patterns look similar with respect to peak broadening and relative intensities between diffraction peaks. The exception is the broad feature due to scattering from the solvent and solutes observed for SBN40_T300_in. From the SEM images cube shaped particles are observed for both setups, where slightly smaller sizes are observed for SBN40_T300_1h compared with SBN40_T300_in. Also, some dimple-like features can be observed for SBN40_T300_1h.

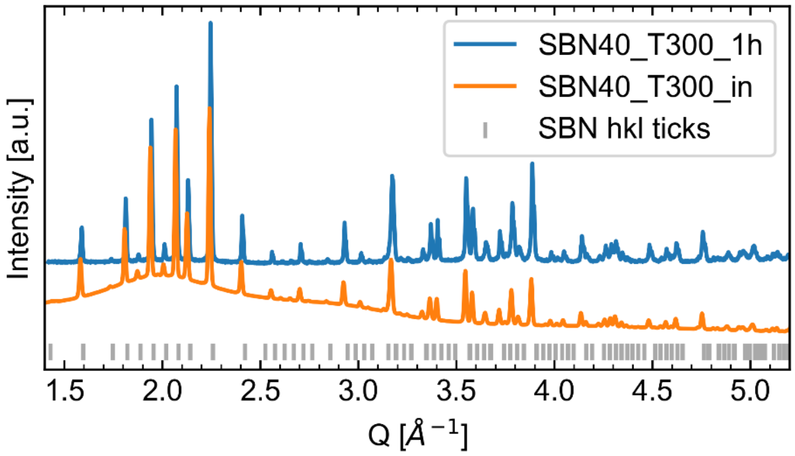

Figure S1: XRD of SBN40_T300_1h and SBN40_T300_0.6h from the coil and *in situ* setup respectively. Both are phase pure SBN. The broad feature observed for SBN40_T300_0.6h is due to scattering from solvent and solutes.

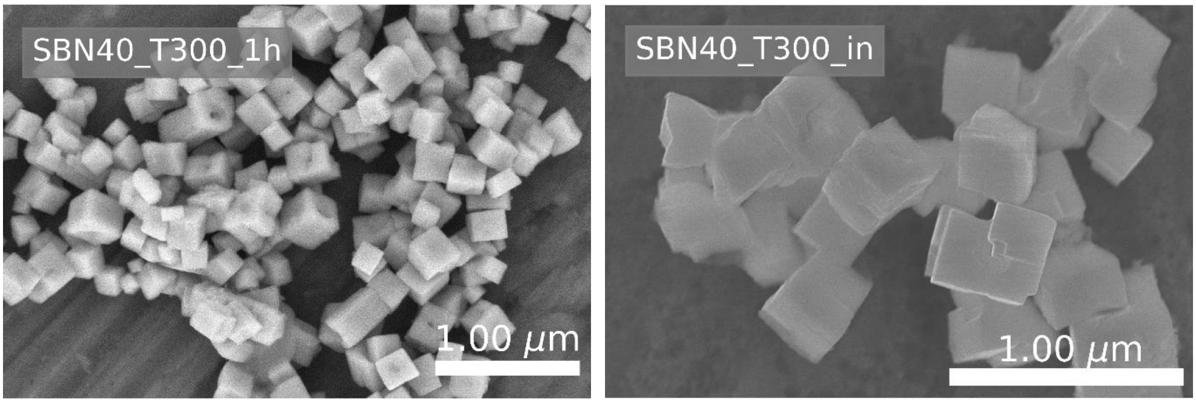

Figure S2: SEM images of SBN40_T300_1h and SBN40_T300_in from the coil and *in situ* setup, respectively. Both setups give cube shaped particles with a cross-sections of ~ 500 x 500 nm^2.

XRD patterns and PDF data for the coil synthesis experiments of SBN

In Figure S3 XRD patterns of the products synthesized using the coil are presented. The XRD patterns show phase pure SBN. For SBN40_T200_1h, the two broad features show the presence of amorphous material because the reaction is not complete after 1 h at 200 °C. The PDF data fitted with a structural model of SBN for all the fully crystalline materials are presented in Figure S4.

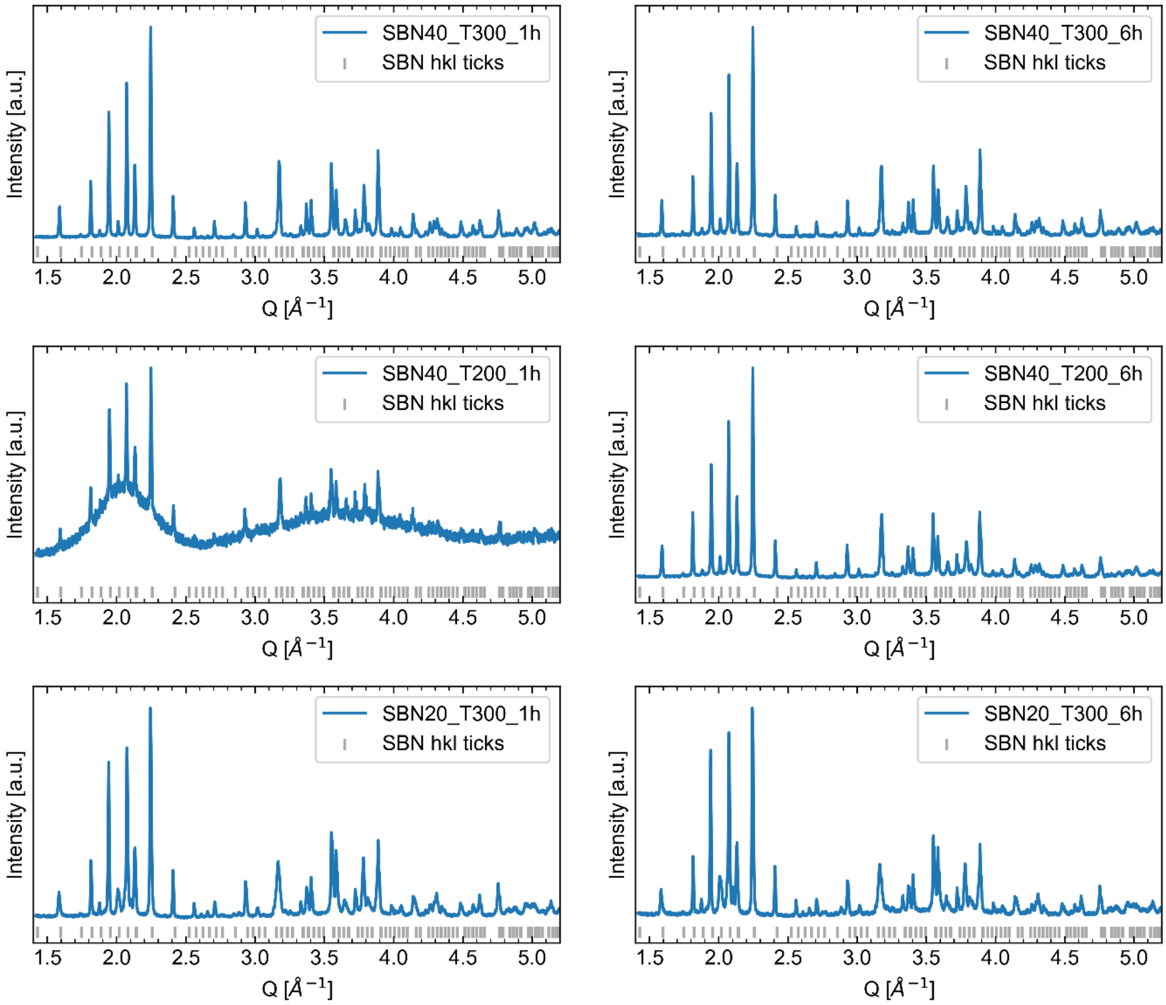

Figure S3: XRD patterns of the products from the coil experiments conducted. All the XRD patterns show phase pure SBN, with SBN40_T200_1h also having two broad features due to amorphous material.

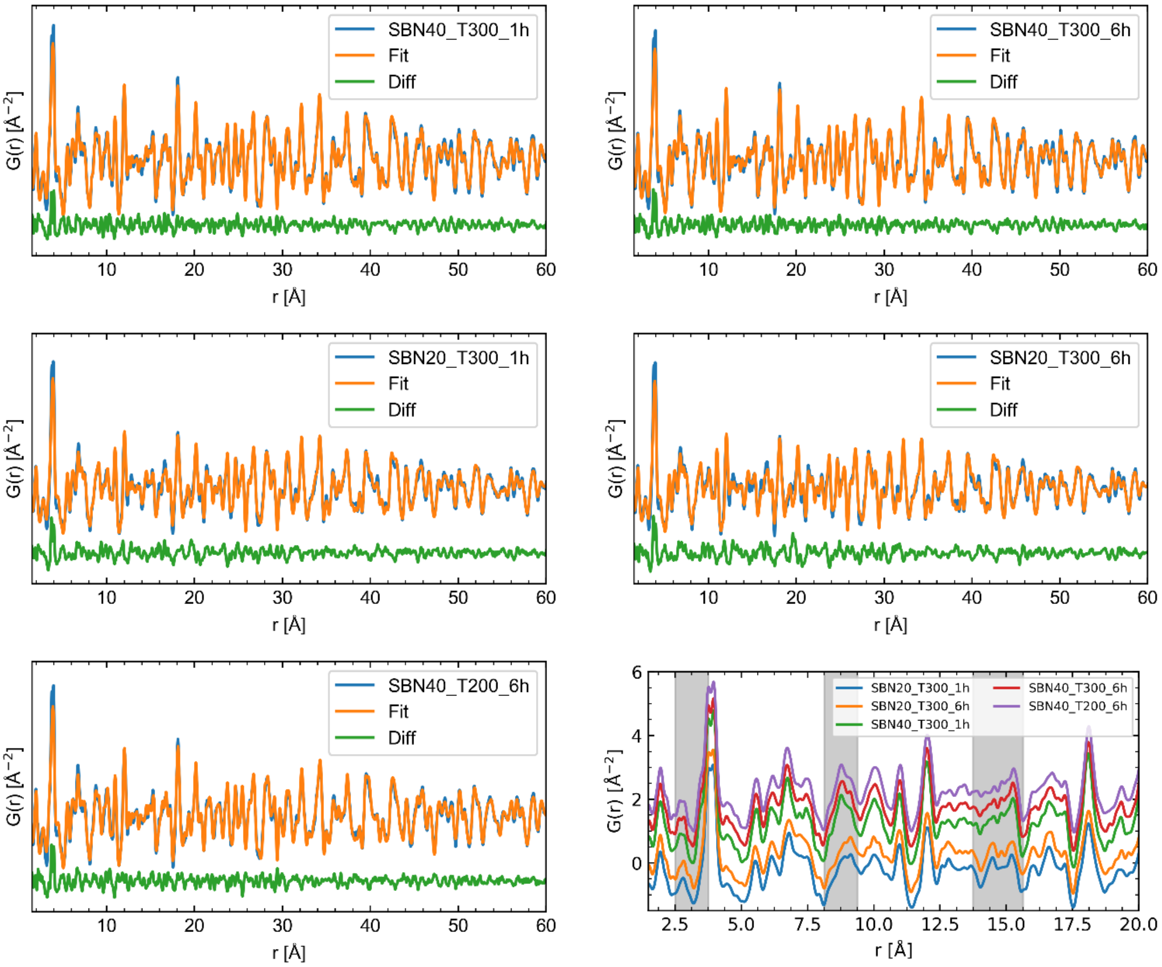

Figure S4: PDF data fitted with a structural model of SBN for the coil syntheses where only crystalline material was formed. Data is plotted in blue, fit to the data in orange and the difference in green. A zoom-in on the low r-region (1.5 -20 Å) with gray areas indicting the most prominent differences in the local structure between SBN20 and SBN40 experiments are also presented.

The structural model used for SBN is as described in [1]. The only difference to this work is the use of the non-centrosymmetric (no. 100, P4bm) instead of the centrosymmetric unit cell (no. 127 P4/mbm). For the refinements scale factor, two lattice parameters (a and c in the tetragonal cell), a spherical size parameter, isotropic APD for both niobium (B1 and B2-sites) and alkaline earth (A1- and A2-sites) sites (4 in total), Sr fraction, Sr occupancy on the A1-site and three atomic position parameters (in total 14 structural parameters). The three atomic position parameters were the x and y parameter for the B2- and A1-sites, respectively (only one needed for the A1-site due to symmetry constraints). The structure reported by Carrio et al. [2] was used as a starting point, and also locking all non-refined ADP (e.g. oxygen) values to the ones obtained with neutron diffraction. Sr was allowed to occupy both A1- and A2-sites while Ba was locked to the A2-site, all while keeping physical meaningful occupancies (*e.g.* not negative or higher than 1) and the sum of Sr and Ba equal to 5 for a unit cell.

Table S1: Structural parameters (scale factor, Sr-fraction, lattice parameters *a* and *c*, ADP values for niobium [B1 and B2] and alkali earth [A1 and A2] and R_{wp}) obtained from PDF analysis of the samples prepared in the coil setup.

Name	Scale factor	Sr-fraction	*a* [Å]	*c* [Å]	ADP [Å^2] B1	ADP [Å^2] B2	ADP [Å^2] A1	ADP [Å^2] A2	R_{wp} [%]
SBN40_T300_1h	0.575(2)	0.35(1)	12.5099(2)	3.9663(1)	1.21(2)	0.621(8)	0.40(2)	1.31(2)	20
SBN40_T300_6h	0.548(2)	0.35(1)	12.5170(2)	3.9687(1)	1.23(2)	0.622(8)	0.41(2)	1.34(2)	21
SBN40_T200_6h	0.543(2)	0.33(1)	12.5318(3)	3.9651(2)	1.23(2)	0.621(9)	0.38(2)	1.55(2)	22
SBN20_T300_1h	0.485(2)	0.25(1)	12.5056(3)	3.9785(2)	1.21(2)	0.622(9)	0.28(3)	1.37(2)	24
SBN20_T300_6h	0.471(2)	0.24(2)	12.5057(3)	3.9803(2)	1.20(3)	0.620(11)	0.25(3)	1.37(2)	28

Bright field (BF) TEM and crystallographic orientation of hollow-ended SBN nanostructures

TEM BF images are presented in Figure S5 and HAADF-STEM and electron diffraction pattern and in Figure S6. Since the two diffraction patterns are taken along two different zone axes, we show that this arises from a relative tilt between the two particles, and not any differences in the crystallographic direction in the particles. Assuming the particle with the [110] zone axis is viewed normal to a facet, we would assume a box-function for the intensity across the particle (bottom schematic in Figure S6 e)), which is what we see in Figure S6 c). Assuming the particle with the [210] zone axis is slightly rotated around its long axis, we would expect an intensity profile as schematically shown at the top of Figure S6 e), which is what we see for the particle in Figure S6 a). From the intensity profile, the angle of tilt can be calculated by combining the following equations when assuming a square cross section, where θ is the tilt angle, and a, b, and c are as shown in Figure S6 f);

$$b = c - 2a * \sin(\theta)$$

$$c = a * \sin(\theta) + a * \cos(\theta)$$

From the values shown in Figure S6 e), θ = 17.6°, which is in good agreement with the theoretical angle of 18.4° between the [110] and [210] direction.

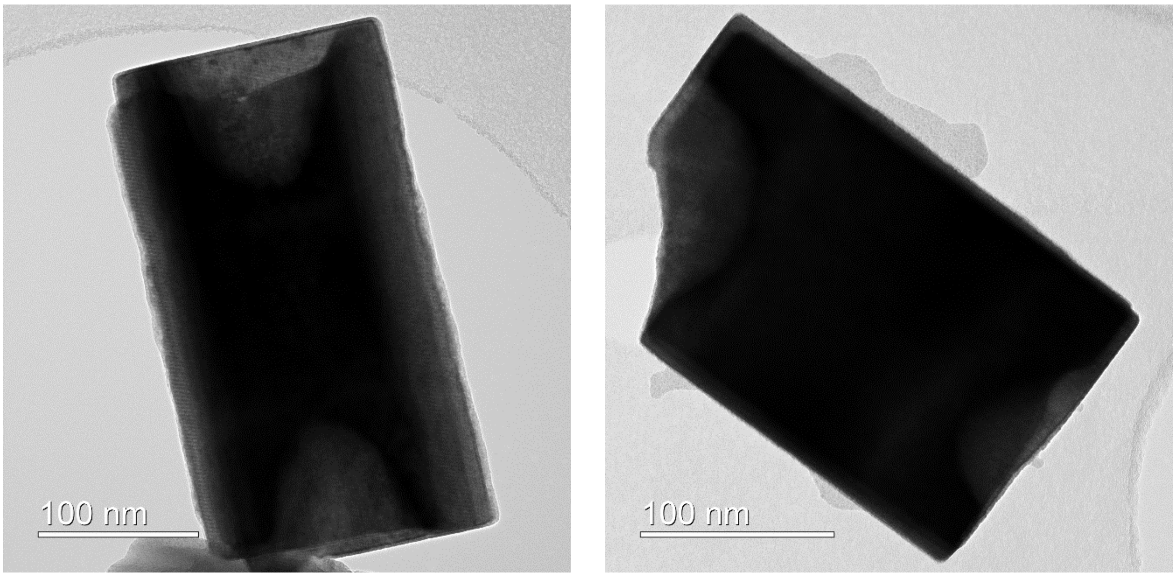

Figure S5: Bright field TEM of the two particles from SBN20_T300_1h characterized with HAADF-STEM and EDS.

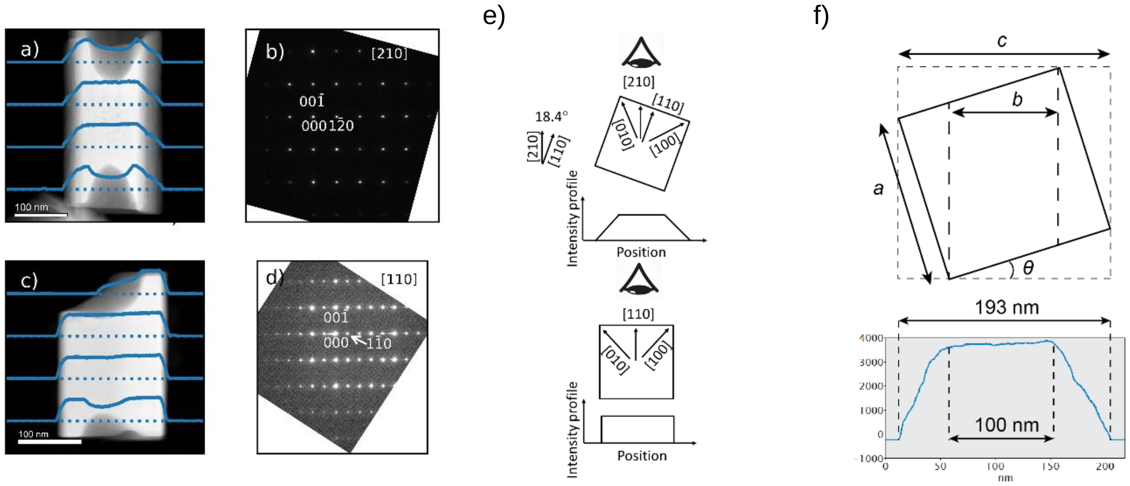

Figure S6: a) and c) HAADF-STEM images of two hollow ended rods from SBN20_T300_1h with blue lines showing the intensity profiles along the blue dashed lines. b) and d) Indexed electron diffraction patterns for the rods are presented in a) and d) respectively. e) Relationship between the [110] and [210]-zone axis, with hypothetical intensity profiles with the [001]-direction pointing into the plane. f) schematic showing how the tilt angle between the two rods were calculated and the determination of values for *b* and *c* from one intensity profile.

References

[1] O. G. Grendal, A. B. Blichfeld, T. D. Vu, W. van Beek, S. M. Selbach, T. Grande, M.-A. Einarsrud, *CrystEngComm* **2019**, *21*, 5922-5930.

[2] J. G. Carrio, Y. P. Mascarenhas, W. Yelon, I. A. Santos, D. Garcia, J. A. Eiras, *Mater. Res* **2002**, *5,* 57-62.
